# Effects of Dietary Betaine on Growth Performance, Serum Metabolites, and Meat Quality of Pigs: A Meta-Analysis

**DOI:** 10.3390/ani16121883

**Published:** 2026-06-17

**Authors:** Guanzu Liu, Yuxuan Wang, Xinyang Dong, Haichao Wang

**Affiliations:** 1 School of Life Sciences and Food Engineering, Hebei University of Engineering, Handan 056038, China; liugz@zju.edu.cn (G.L.);; 2 College of Animal Sciences, Zhejiang University, Hangzhou 310058, China

**Keywords:** betaine, swine, growth performance, meat quality, meta-analysis

## Abstract

Betaine, functioning as a key methyl donor and organic osmolyte, is widely used to improve growth performance and carcass characteristics of swine. However, existing studies show inconsistent results, and the optimal supplementation dose remains unclear. This study systematically quantified the impact of betaine supplementation on the growth performance, serum metabolites, and meat quality of swine via a meta-analysis and meta-regression of 31 randomized controlled trials. The standardized mean differences (SMDs) were calculated using a random-effects model, while a subgroup analysis and meta-regression were used to investigate potential sources of heterogeneity. The analysis revealed that dietary betaine supplementation significantly increased the average daily gain and reduced the feed conversion ratio, while the average daily feed intake remained unaffected. A subgroup analysis indicated that the optimal dose for improving feed conversion was approximately 1250 mg/kg, whereas this efficacy was attenuated in the high-dose cohorts. Furthermore, betaine significantly reduced serum urea levels, muscle shear force, drip loss, and cooking loss. Conversely, the backfat thickness and serum lipid indices showed no significant changes. The meta-regression confirmed that the breed and growth stage were the primary determinants of heterogeneity in growth performance. These findings demonstrate that betaine facilitates growth primarily by enhancing feed efficiency and protein accretion, and its beneficial effects on meat quality are mainly driven by improved tenderness and water-holding capacity.

## Simple Summary

Betaine, acting as a key methyl donor and organic osmolyte, is widely used to improve growth performance and carcass characteristics in swine. However, the findings across studies are inconsistent, and the optimal supplementation dose remains unclear. This meta-analysis investigated the effects of dietary betaine on growth performance, serum metabolites, and meat quality of pigs. The results demonstrated a positive effect of betaine supplementation on the average daily gain and feed conversion efficiency, without significantly altering the feed intake. Furthermore, betaine supplementation improved meat quality by enhancing pork tenderness and water-holding capacity. The analysis also identified a threshold effect, showing an optimal dietary inclusion level of approximately 1250 mg/kg for improving feed conversion, whereas this efficacy was attenuated at higher doses. These promising results indicate that dietary betaine could be effectively utilized as a nutritional strategy to improve swine productivity and pork quality.

## 1. Introduction

Driven by the global shift toward antibiotic-use reduction and sustainable husbandry practices, the investigation into novel feed additives that enhance growth, mitigate stress, and improve meat quality has become a focal point of contemporary research [1]. Currently, the swine industry faces a dual challenge. On the one hand, producers must maintain rapid growth rates and optimal feed conversion efficiency [2]. On the other hand, evolving consumer preferences are driving an escalating demand for premium pork, characterized by a high lean yield and reduced fat deposition [3]. Consequently, the optimization of carcass attributes, concomitant with the preservation of growth performance, constitutes the crux of contemporary nutritional intervention strategies.

Betaine, a natural trimethylglycine that widely exists in living beings, demonstrates considerable utility in swine nutrition due to its distinct physiological mechanisms [4]. Primarily acting as a potent methyl donor, betaine facilitates the methylation of homocysteine, which exerts a methionine-sparing effect while simultaneously promoting protein accretion [5,6]. Secondly, betaine modulates the key enzymes involved in lipid metabolism to induce energy repartitioning, thereby mitigating backfat thickness and optimizing lean carcass yield [7,8]. Furthermore, functioning as a critical organic osmolyte, betaine maintains osmotic homeostasis within enterocytes, which reinforces intestinal barrier integrity in swine compromised by weaning transition or environmental stress [6,9].

Despite its well-established theoretical mechanisms, the empirical effects of betaine on swine growth performance and pork quality remain controversial in production settings [10,11,12,13]. Numerous studies have supported the growth-promoting efficacy of betaine. For instance, Siljander-Rasi et al. [10] observed that dietary supplementation with 0.1% betaine linearly improved both the average daily gain (ADG) and feed conversion ratio (FCR). On the contrary, other investigations have indicated that betaine supplementation fails to improve growth performance [11,12]. Certain studies have even reported adverse effects under specific experimental conditions [13].

The existing evidence suggests that the efficacy of betaine is modulated by multiple factors, including the supplement dose, genetic background, basal dietary composition, and growth phase. However, a systematic quantitative assessment addressing these potential confounding variables is currently lacking. Furthermore, the specific dose–response relationship required to establish optimal inclusion levels in practice is yet to be determined. Therefore, the present study leveraged meta-analysis and meta-regression methodologies to comprehensively evaluate the impact of betaine on the growth performance, serum metabolic profiles, and carcass traits of swine, thereby providing a robust scientific basis for precision nutrition strategies in swine husbandry.

## 2. Materials and Methods

### 2.1. Literature Search Strategy

This study was conducted in strict adherence to the Preferred Reporting Items for Systematic Reviews and Meta-Analyses (PRISMA) guidelines. A systematic literature search was performed across PubMed, ScienceDirect, and Web of Science for articles published between January 2001 and December 2025. The search syntax combined the following subject terms (“pig” OR “swine” OR “porcine” OR “piglet” OR “finishing pig”) and the intervention term (“betaine”). The detailed search strategy and the results are presented in Table 1.

### 2.2. Inclusion and Exclusion Criteria

The study selection was based on the PICOS framework (Participants, Interventions, Comparators, Outcomes, and Study Design): (1) Participants: Healthy swine, including weaned piglets and growing–finishing pigs; (2) Interventions: Dietary supplementation of betaine with clearly defined doses in the experimental group (regardless of chemical form); (3) Comparators: A control group fed a basal diet without antibiotics or betaine, with clearly defined dose parameters; (4) Outcomes: Studies reporting at least one of the following parameters: average daily gain (ADG), average daily feed intake (ADFI), feed conversion ratio (FCR), meat quality traits (e.g., meat color L*, a*, b*, backfat thickness), or serum metabolites (e.g., urea, TG); and (5) Study Design: Randomized controlled trials.

The exclusion criteria were established as follows: (1) Publication Type: Reviews, meta-analyses, or non-English articles; (2) Species: Studies involving murine models or other non-porcine species; (3) Confounded Interventions: Experimental designs where betaine was not the sole variable (i.e., combined interventions); (4) Atypical Conditions: Studies utilizing low-protein diets or abnormal rearing environments; (5) Insufficient Data: Failure to report sample size or replicates, incomplete outcome data (e.g., absence of mean values or SD/SEM), clear statistical errors or logical inconsistencies, or duplicate publications utilizing identical datasets.

### 2.3. Data Extraction

Two investigators (G.Z. Liu and Y.X. Wang) independently extracted the data using a standardized protocol. The extracted results were cross-checked for accuracy, and any discrepancies were resolved through discussion or by consulting a third investigator (H.C. Wang). For studies evaluating multiple betaine doses against a single control, only one treatment group was extracted based on data completeness or via random selection when completeness was equivalent.

The following data were extracted: (1) Bibliographic Information: First author and publication year; (2) Animal Characteristics: breed, sex, initial body weight, and growth stage; (3) Intervention Details: Sample size, specific betaine dose (mg/kg), and basal diet composition; (4) Outcome Measures: Means, standard deviations (SDs), or standard errors of the mean (SEM) for growth performance, serum biochemistry, and meat quality indices across treatment groups.

### 2.4. Data Standardization and Processing

To ensure analytical homogeneity, the extracted data were pre-processed as follows: (1) Variance Conversion: For studies reporting only the standard error of the mean (SEM), the standard deviation (SD) was calculated using the formula SD = SEM × sqrt(n). If only a pooled SEM was provided, equal variance across treatment groups was assumed to estimate the group-specific SD; (2) Units Standardization: All outcome metrics were converted to the International System of Units (SI) to ensure data consistency and to facilitate the preliminary screening of potential outliers.

### 2.5. Statistical Analysis

#### 2.5.1. Meta-Analysis Framework

The statistical analyses were executed utilizing the R statistical environment (Version 4.5.2; R Foundation for Statistical Computing, Vienna, Austria) in conjunction with the companion ‘meta’ package (Version 8.2-1). Because the measurement units and assay methods varied across studies, the effect sizes for continuous variables were expressed as standardized mean differences (SMDs) with 95% confidence intervals (CIs).

To account for the expected heterogeneity from the differences between genetic backgrounds, feeding regimes, and basal diets, a random-effects model was applied to all the meta-analyses. Study weights were assigned using the inverse-variance method, and the restricted maximum likelihood (REML) estimator was used to calculate between-study variance.

#### 2.5.2. Exploration of Heterogeneity

To explore potential sources of heterogeneity, a dual approach combining meta-regression and subgroup analyses were adopted. In the meta-regression models, the betaine doses, swine breeds, and experimental phases were included as covariates. Specifically, the betaine dose was included as a continuous covariate, while the growth phase and breed were converted into nominal variables. The growing–finishing phase and Duroc × Landrace × Yorkshire (DLY) pigs were set as the reference baselines. Furthermore, subgroup analyses were performed based on predefined criteria whenever significant heterogeneity was detected (*p* < 0.10 or I^2^ > 50%).

#### 2.5.3. Heterogeneity Assessment

Inter-study heterogeneity was qualitatively assessed via Cochran’s Q test (statistical significance defined as *p* < 0.10) and quantitatively characterized using the I^2^ statistic. In accordance with Higgins’ classification, I^2^ values approximating 25%, 50%, and 75% were interpreted as indicative of low, moderate, and high heterogeneity, respectively. An I^2^ > 50% threshold signified substantial heterogeneity, warranting focused investigation into its origins.

#### 2.5.4. Sensitivity Analysis and Publication Bias

The robustness of the meta-analytic results was evaluated using a leave-one-out sensitivity analysis. This involved iteratively excluding individual studies and recalculating the pooled effect sizes to determine if the overall results were significantly altered. The publication bias was visually assessed using funnel plots. The plot symmetry was further evaluated statistically using Egger’s regression test and Begg’s rank correlation test. Egger’s test was performed only when the analysis included more than ten studies.

## 3. Results

### 3.1. Literature Search Results

**Figure 1:**
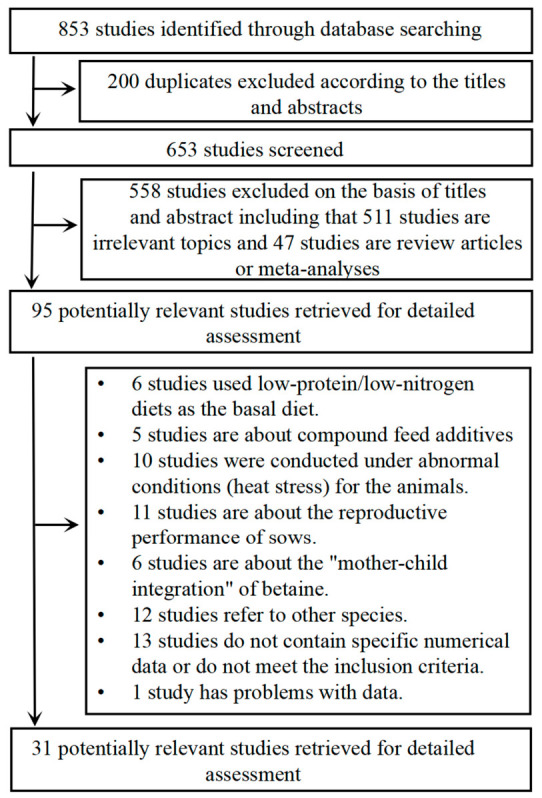
Study selection process.

The systematic literature search across PubMed, Web of Science, and ScienceDirect yielded a total of 853 citations published between January 2001 and December 2025. After removing 200 duplicates, the remaining records were screened by title and abstract, resulting in the exclusion of 511 irrelevant articles. A subsequent full-text assessment excluded an additional 47 reviews and 64 studies that did not meet the inclusion criteria. Ultimately, 31 randomized controlled trials (RCTs) were included in the quantitative synthesis. Among the included studies, the dataset comprised three trials focusing on weaned piglets and 28 on growing–finishing pigs. The detailed characteristics of the included literature are summarized in Figure 1.

### 3.2. Characteristics of Included Studies

The studies included in this meta-analysis were published between 2001 and 2025. The dietary betaine supplementation levels ranged from 500 to 5000 mg/kg. Regarding the growth phase of the animals, the trials primarily involved two distinct stages: weaned piglets and growing–finishing pigs. The detailed characteristics of the included studies are presented in Table 2.

### 3.3. Meta-Analysis of Growth Performance

**Figure 2:**
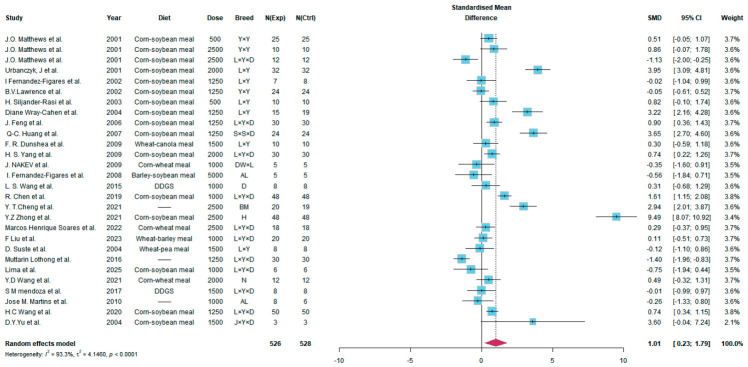
Forest plot of the overall effect of betaine supplementation on pigs’ ADG. The plot displays the individual and pooled effect sizes, expressed as standardized mean differences (SMDs) with their corresponding 95% confidence intervals (CIs). The size of each square is proportional to the study’s statistical weight in the overall estimate. The solid vertical black line represents an SMD of zero, indicating no effect. Points to the left of the line represent a reduction in the ADG, while points to the right indicate an increase. The horizontal lines connected to the squares represent the 95% CI for individual effect sizes. The overall pooled effect size and its 95% CI are indicated by the diamond at the bottom. This effect is highly heterogeneous, as indicated by an I^2^ of 93.3%. The blue squares represent the effect sizes of individual studies, and the red diamonds represent the pooled effect sizes [10,11,12,13,14,15,16,17,18,19,20,21,22,23,24,25,26,27,28,29,30,31,32,33,34,35,36,40].

**Figure 3:**
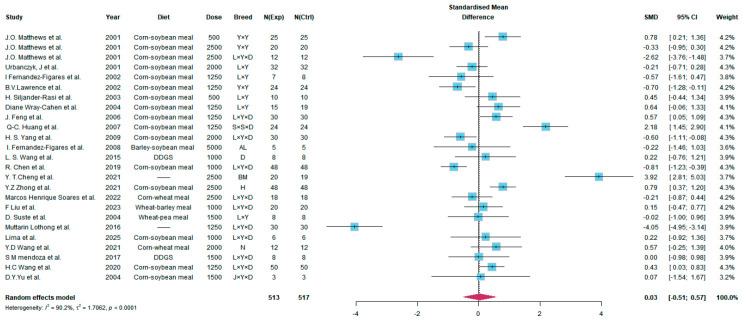
Forest plot of the overall effect of betaine supplementation on pigs’ ADFI. The plot displays the individual and pooled effect sizes, expressed as standardized mean differences (SMDs) with their corresponding 95% confidence intervals (CIs). The size of each square is proportional to the study’s statistical weight in the overall estimate. The solid vertical black line represents an SMD of zero, indicating no effect. Points to the left of the line represent a reduction in the ADFI, while points to the right indicate an increase. The horizontal lines connected to the squares represent the 95% CI for individual effect sizes. The overall pooled effect size and its 95% CI are indicated by the diamond at the bottom. This effect is highly heterogeneous, as indicated by an I^2^ of 90.2%. The blue squares represent the effect sizes of individual studies, and the red diamonds represent the pooled effect sizes [10,11,13,14,15,16,17,18,19,20,21,22,24,25,26,27,28,29,30,31,32,33,34,36,40].

**Figure 4:**
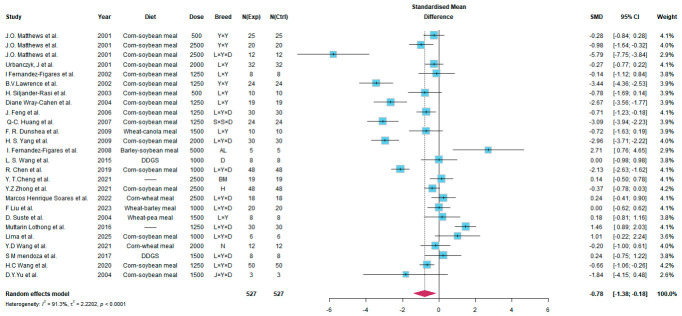
Forest plot of the overall effect of betaine supplementation on pigs’ FCR. The plot displays the individual and pooled effect sizes, expressed as standardized mean differences (SMDs) with their corresponding 95% confidence intervals (CIs). The size of each square is proportional to the study’s statistical weight in the overall estimate. The solid vertical black line represents an SMD of zero, indicating no effect. Points to the left of the line represent a reduction in the FCR, while points to the right indicate an increase. The horizontal lines connected to the squares represent the 95% CI for individual effect sizes. The overall pooled effect size and its 95% CI are indicated by the diamond at the bottom. This effect is highly heterogeneous, as indicated by an I^2^ of 91.3%. The blue squares represent the effect sizes of individual studies, and the red diamonds represent the pooled effect sizes [10,11,13,14,15,16,17,18,19,20,21,22,23,24,25,26,27,28,29,30,31,32,33,34,36,40].

The meta-analytic results concerning the impact of dietary betaine on swine ADG, ADFI, and FCR are summarized in Table 3, Table 4 and Table 5. The aggregate data indicate that betaine supplementation conferred a significant ameliorative effect on the overall growth performance. Specifically, the betaine-treated cohort exhibited a significant increase in the ADG [SMD = 1.01; 95% CI: (0.23, 1.79); *p* < 0.05] (Figure 2), while no significant effect was observed on the ADFI [SMD = 0.03; 95% CI: (−0.51, 0.57); *p* > 0.05] (Figure 3). Consequently, these dynamics contributed to a significant reduction in the FCR [SMD = −0.78; 95% CI: (−1.38, −0.18); *p* < 0.05] (Figure 4). Among the evaluated parameters, the ADG demonstrated the most substantial overall improvement. The heterogeneity assessments revealed that the ADG, ADFI, and FCR were all subject to pronounced heterogeneity (I^2^ > 90%), with the ADG exhibiting the highest level of variance, indicating severe inconsistency across the included studies and thereby warranting further exploration into the sources of variance.

### 3.4. Subgroup Analysis and Meta-Regression

To explore the sources of the substantial heterogeneity in growth performance, subgroup analyses (Table 3, Table 4 and Table 5) and meta-regressions (Table 6), stratified by the growth phase, betaine dose and swine breed, were conducted.

**Figure A1:**
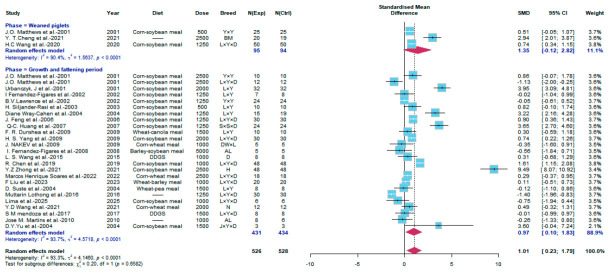
Subgroup forest plots of the effect of betaine supplementation on ADG across pig phases. The forest plots illustrate the subgroup analyses of betaine supplementation effects. The effect sizes are expressed as standardized mean differences (SMDs) with corresponding 95% confidence intervals (CIs). The size of each square is proportional to the study’s statistical weight. The solid vertical black line represents an SMD of zero (no effect). Points to the left of the line represent a reduction in the outcome metric, while points to the right indicate an increase. The overall pooled effect size for each subgroup and its 95% CI are indicated by the diamonds at the bottom. The blue squares represent the effect sizes of individual studies, and the red diamonds represent the pooled effect sizes [10,11,12,13,14,15,16,17,18,19,20,21,22,23,24,25,26,27,28,29,30,31,32,33,34,35,36,40].

**Figure A2:**
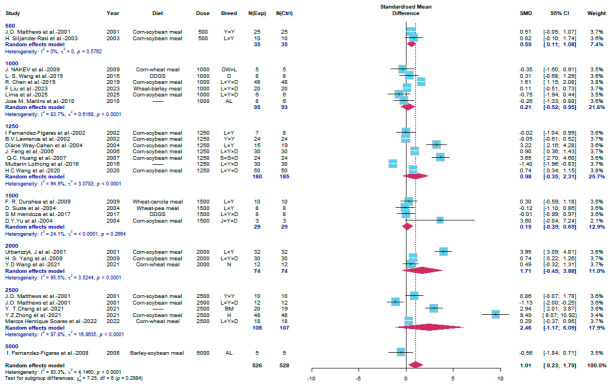
Subgroup forest plots of the effect of betaine supplementation dose on pigs’ ADG. The forest plots illustrate the subgroup analyses of betaine supplementation effects. The effect sizes are expressed as standardized mean differences (SMDs) with corresponding 95% confidence intervals (CIs). The size of each square is proportional to the study’s statistical weight. The solid vertical black line represents an SMD of zero (no effect). Points to the left of the line represent a reduction in the outcome metric, while points to the right indicate an increase. The overall pooled effect size for each subgroup and its 95% CI are indicated by the diamonds at the bottom. The blue squares represent the effect sizes of individual studies, and the red diamonds represent the pooled effect sizes [10,11,12,13,14,15,16,17,18,19,20,21,22,23,24,25,26,27,28,29,30,31,32,33,34,35,36,40].

**Figure A3:**
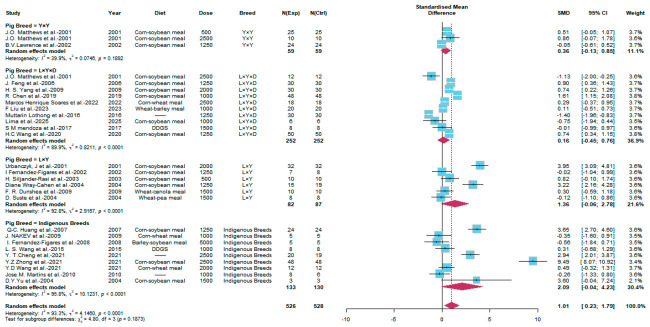
Subgroup forest plots of the effect of betaine supplementation on ADG across pig breeds. The forest plots illustrate the subgroup analyses of betaine supplementation effects. The effect sizes are expressed as standardized mean differences (SMDs) with corresponding 95% confidence intervals (CIs). The size of each square is proportional to the study’s statistical weight. The solid vertical black line represents an SMD of zero (no effect). Points to the left of the line represent a reduction in the outcome metric, while points to the right indicate an increase. The overall pooled effect size for each subgroup and its 95% CI are indicated by the diamonds at the bottom. The blue squares represent the effect sizes of individual studies, and the red diamonds represent the pooled effect sizes [10,11,12,13,14,15,16,17,18,19,20,21,22,23,24,25,26,27,28,29,30,31,32,33,34,35,36,40].

Regarding the ADG, the physiological growth phase significantly influenced betaine efficacy. Specifically, a significant improvement in ADG was observed in the growing–finishing pigs [SMD = 0.97; 95% CI: (0.10, 1.83); *p* < 0.05] (Figure A1). In contrast, the subgroup analysis showed that neither the betaine dose nor breed significantly affected ADG (Figure A2 and Figure A3). However, the meta-regression identified the breed as the primary source of variance for ADG (*p* < 0.05).

**Figure A4:**
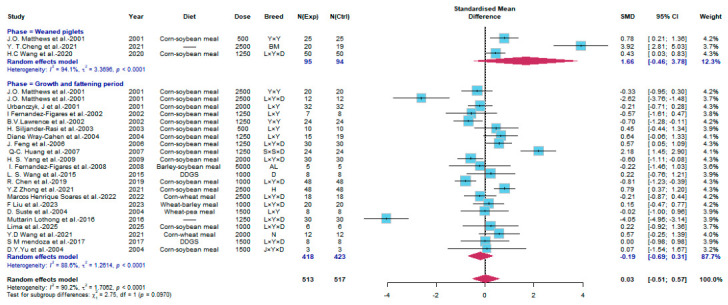
Subgroup forest plots of the effect of betaine supplementation on ADFI across pig phases. The forest plots illustrate the subgroup analyses of betaine supplementation effects. The effect sizes are expressed as standardized mean differences (SMDs) with corresponding 95% confidence intervals (CIs). The size of each square is proportional to the study’s statistical weight. The solid vertical black line represents an SMD of zero (no effect). Points to the left of the line represent a reduction in the outcome metric, while points to the right indicate an increase. The overall pooled effect size for each subgroup and its 95% CI are indicated by the diamonds at the bottom. The blue squares represent the effect sizes of individual studies, and the red diamonds represent the pooled effect sizes [10,11,13,14,15,16,17,18,19,20,21,22,24,25,26,27,28,29,30,31,32,33,34,36,40].

**Figure A5:**
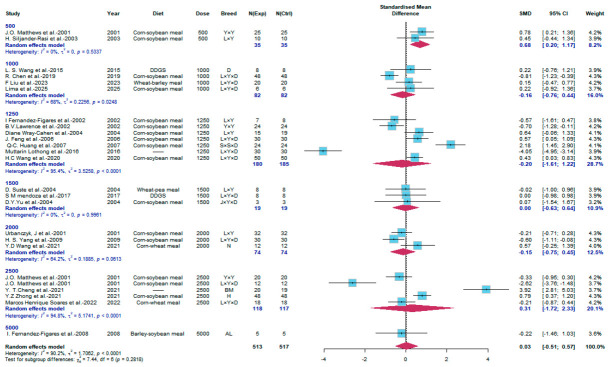
Subgroup forest plots of the effect of betaine supplementation dose on pigs’ ADFI. The forest plots illustrate the subgroup analyses of betaine supplementation effects. The effect sizes are expressed as standardized mean differences (SMDs) with corresponding 95% confidence intervals (CIs). The size of each square is proportional to the study’s statistical weight. The solid vertical black line represents an SMD of zero (no effect). Points to the left of the line represent a reduction in the outcome metric, while points to the right indicate an increase. The overall pooled effect size for each subgroup and its 95% CI are indicated by the diamonds at the bottom. The blue squares represent the effect sizes of individual studies, and the red diamonds represent the pooled effect sizes [10,11,13,14,15,16,17,18,19,20,21,22,24,25,26,27,28,29,30,31,32,33,34,36,40].

**Figure A6:**
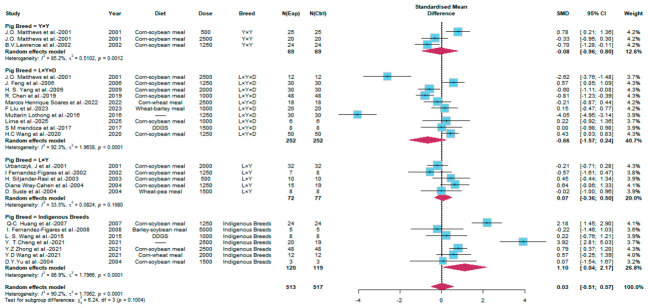
Subgroup forest plots of the effect of betaine supplementation on ADFI across pig breeds. The forest plots illustrate the subgroup analyses of betaine supplementation effects. The effect sizes are expressed as standardized mean differences (SMDs) with corresponding 95% confidence intervals (CIs). The size of each square is proportional to the study’s statistical weight. The solid vertical black line represents an SMD of zero (no effect). Points to the left of the line represent a reduction in the outcome metric, while points to the right indicate an increase. The overall pooled effect size for each subgroup and its 95% CI are indicated by the diamonds at the bottom. The blue squares represent the effect sizes of individual studies, and the red diamonds represent the pooled effect sizes [10,11,13,14,15,16,17,18,19,20,21,22,24,25,26,27,28,29,30,31,32,33,34,36,40].

For the ADFI, the overall effect of betaine remained statistically insignificant across the different growth phases and doses (Figure A4 and Figure A5). The subgroup analysis revealed that betaine significantly increased the ADFI specifically in indigenous breeds [SMD = 1.10; 95% CI: (0.04, 2.17); *p* < 0.05] (Figure A6). Furthermore, the meta-regression indicated that both the breed (*p* < 0.05) and growth phase (*p* < 0.05) were significantly correlated with the effect sizes. These two factors accounted for the majority of the variance in the ADFI, highlighting that sensitivity to betaine varied depending on the genetic background and physiological status.

**Figure A7:**
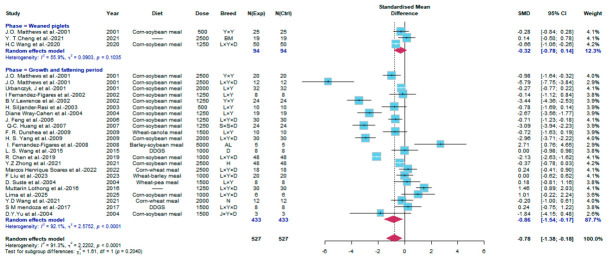
Subgroup forest plots of the effect of betaine supplementation on FCR across pig phases. The forest plots illustrate the subgroup analyses of betaine supplementation effects. The effect sizes are expressed as standardized mean differences (SMDs) with corresponding 95% confidence intervals (CIs). The size of each square is proportional to the study’s statistical weight. The solid vertical black line represents an SMD of zero (no effect). Points to the left of the line represent a reduction in the outcome metric, while points to the right indicate an increase. The overall pooled effect size for each subgroup and its 95% CI are indicated by the diamonds at the bottom. The blue squares represent the effect sizes of individual studies, and the red diamonds represent the pooled effect sizes [10,11,13,14,15,16,17,18,19,20,21,22,23,24,25,26,27,28,29,30,31,32,33,34,36,40].

**Figure A8:**
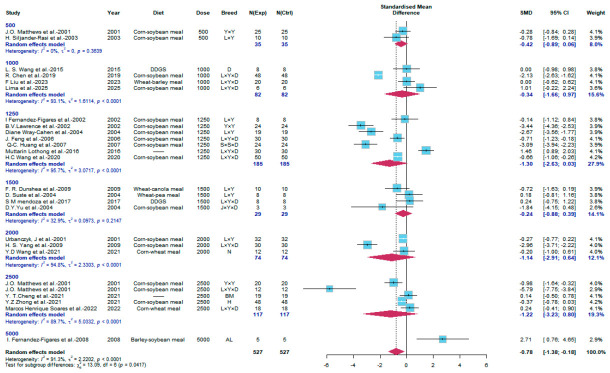
Subgroup forest plots of the effect of betaine supplementation dose on pigs’ FCR. The forest plots illustrate the subgroup analyses of betaine supplementation effects. The effect sizes are expressed as standardized mean differences (SMDs) with corresponding 95% confidence intervals (CIs). The size of each square is proportional to the study’s statistical weight. The solid vertical black line represents an SMD of zero (no effect). Points to the left of the line represent a reduction in the outcome metric, while points to the right indicate an increase. The overall pooled effect size for each subgroup and its 95% CI are indicated by the diamonds at the bottom. The blue squares represent the effect sizes of individual studies, and the red diamonds represent the pooled effect sizes [10,11,13,14,15,16,17,18,19,20,21,22,23,24,25,26,27,28,29,30,31,32,33,34,36,40].

**Figure A9:**
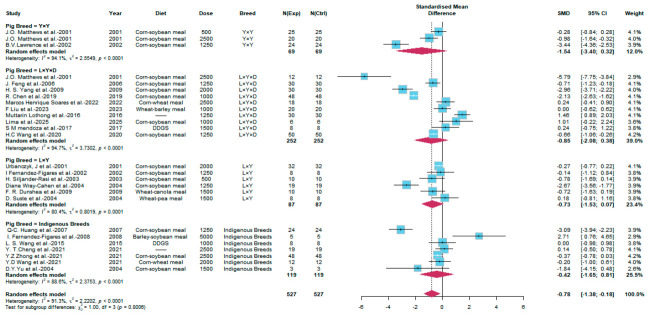
Subgroup forest plots of the effect of betaine supplementation on FCR across pig breeds. The forest plots illustrate the subgroup analyses of betaine supplementation effects. The effect sizes are expressed as standardized mean differences (SMDs) with corresponding 95% confidence intervals (CIs). The size of each square is proportional to the study’s statistical weight. The solid vertical black line represents an SMD of zero (no effect). Points to the left of the line represent a reduction in the outcome metric, while points to the right indicate an increase. The overall pooled effect size for each subgroup and its 95% CI are indicated by the diamonds at the bottom. The blue squares represent the effect sizes of individual studies, and the red diamonds represent the pooled effect sizes [10,11,13,14,15,16,17,18,19,20,21,22,23,24,25,26,27,28,29,30,31,32,33,34,36,40].

With regard to the FCR, the growth phase significantly influenced betaine efficacy, with a significant reduction observed during the growing–finishing phase [SMD = −0.86; 95% CI: (−1.54, −0.17); *p* = 0.015] (Figure A7). The subgroup analysis identified the dose as a key modulator. Notably, supplementation at the 1250 mg/kg level exhibited a tendency towards ameliorating the FCR [SMD = −1.30; 95% CI: (−2.63, 0.03); *p* = 0.056], whereas this efficacy was attenuated in the high-dose cohort (>2000 mg/kg) (Figure A8). The meta-regression did not establish a significant linear correlation between the dose and FCR effect size (*p* > 0.05). This supports the existence of an optimal dose threshold rather than a simple linear dose–response relationship. Finally, no significant differences in the FCR were observed across swine breeds (Figure A9).

### 3.5. Serum Metabolites

**Figure A10:**
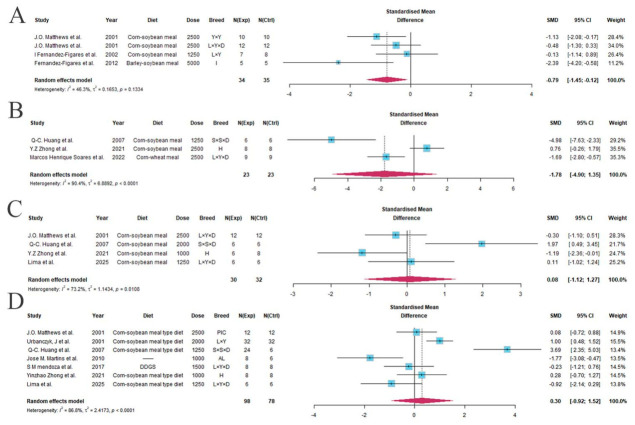
Forest plots of the effect of betaine supplementation on pig serum protein profiles. (**A**) Serum urea; (**B**) serum urea nitrogen (SUN); (**C**) albumin; and (**D**) total protein. The forest plots illustrate the subgroup analyses of betaine supplementation effects. The effect sizes are expressed as standardized mean differences (SMDs) with corresponding 95% confidence intervals (CIs). The size of each square is proportional to the study’s statistical weight. The solid vertical black line represents an SMD of zero (no effect). Points to the left of the line represent a reduction in the outcome metric, while points to the right indicate an increase. The overall pooled effect size for each subgroup and its 95% CI are indicated by the diamonds at the bottom. The blue squares represent the effect sizes of individual studies, and the red diamonds represent the pooled effect sizes [11,13,15,16,17,18,22,29,30,35,37].

**Figure A11:**
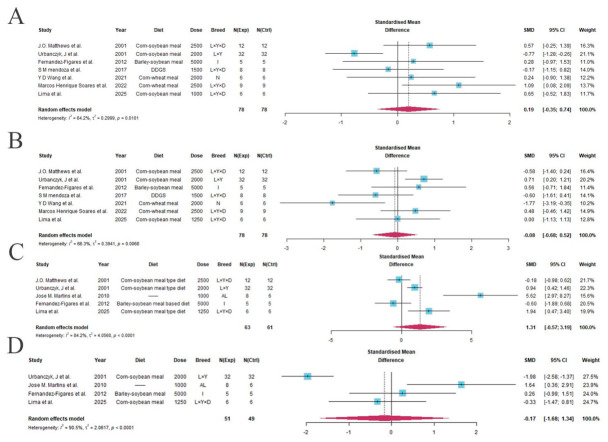
Forest plots of the effect of betaine supplementation on pig serum lipid profiles. (**A**) Total cholesterol (TC); (**B**) triglycerides (TGs); (**C**) high-density lipoprotein (HDL); and (**D**) low-density lipoprotein (LDL). The forest plots illustrate the subgroup analyses of betaine supplementation effects. The effect sizes are expressed as standardized mean differences (SMDs) with corresponding 95% confidence intervals (CIs). The size of each square is proportional to the study’s statistical weight. The solid vertical black line represents an SMD of zero (no effect). Points to the left of the line represent a reduction in the outcome metric, while points to the right indicate an increase. The overall pooled effect size for each subgroup and its 95% CI are indicated by the diamonds at the bottom. The blue squares represent the effect sizes of individual studies, and the red diamonds represent the pooled effect sizes [11,13,16,17,30,34,35,37].

The effects of dietary betaine supplementation on swine serum biochemical indices are summarized in Table 7. The results indicate that betaine significantly affected protein metabolism. Most notably, the serum urea levels were significantly decreased [SMD = −0.79; 95% CI: (−1.45, −0.12); *p* < 0.05] (Figure A10A). This reduction showed low inter-study heterogeneity (I^2^ = 46.3%), indicating a consistent and robust effect across the included studies. Conversely, no significant differences were observed between the treatment and control groups for lipid metabolism indices (triglycerides, total cholesterol, HDL, LDL) or other protein parameters (albumin, total protein) (Figure A10 and Figure A11). Furthermore, these non-significant parameters generally exhibited substantial heterogeneity.

### 3.6. Carcass Traits and Meat Quality

**Figure 5:**
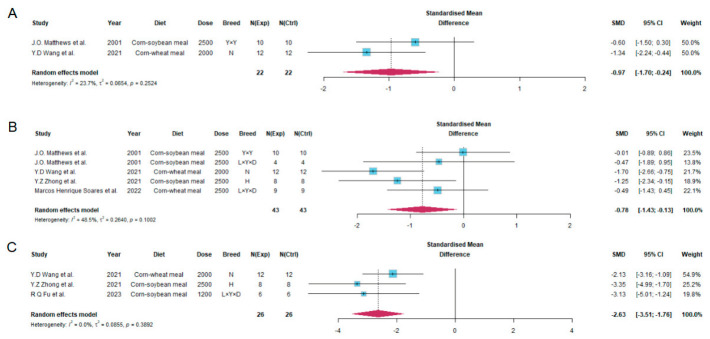
Forest plots demonstrating the effects of dietary betaine supplementation on pork quality. (**A**) Drip loss; (**B**) cooking loss; (**C**) shear force. The effect sizes are expressed as standardized mean differences (SMDs) with corresponding 95% confidence intervals (CIs). The size of each square is proportional to the study’s statistical weight. The solid vertical black line represents an SMD of zero, indicating no effect. Points to the left of the line represent a reduction in the outcome metric, while points to the right indicate an increase. The horizontal lines connected to the squares represent the 95% CIs for individual effect sizes. The overall pooled effect size for each panel and its 95% CI are indicated by the diamonds at the bottom of each panel [15,16,29,30,34,39].

The effects of dietary betaine supplementation on carcass traits and meat quality are summarized in Table 7. Regarding carcass characteristics, betaine demonstrated a significant potential to improve the water-holding capacity (WHC) and tenderness of porcine muscle. Specifically, betaine supplementation significantly reduced both the drip loss [SMD = −0.97; 95% CI: (−1.70, −0.24); *p* < 0.05] and cooking loss [SMD = −0.78; 95% CI: (−1.43, −0.13); *p* < 0.05] (Figure 5) compared to the control group. The I^2^ values associated with these parameters were 23.7% and 48.5%, respectively, indicating a relatively robust efficacy at enhancing the WHC.

**Figure A12:**
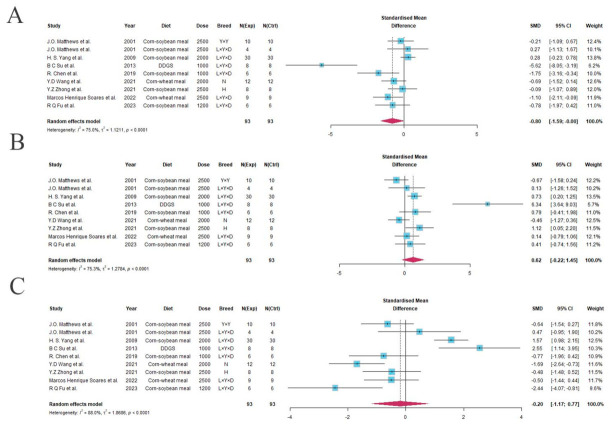
Forest plots of the effect of betaine supplementation on pork color parameters. (**A**) L*; (**B**) a*; (**C**) b*. The forest plots illustrate the subgroup analyses of betaine supplementation effects. The effect sizes are expressed as standardized mean differences (SMDs) with corresponding 95% confidence intervals (CIs). The size of each square is proportional to the study’s statistical weight. The solid vertical black line represents an SMD of zero (no effect). Points to the left of the line represent a reduction in the outcome metric, while points to the right indicate an increase. The overall pooled effect size for each subgroup and its 95% CI are indicated by the diamonds at the bottom. The blue squares represent the effect sizes of individual studies, and the red diamonds represent the pooled effect sizes [15,16,24,27,29,30,34,38,39].

**Figure A13:**
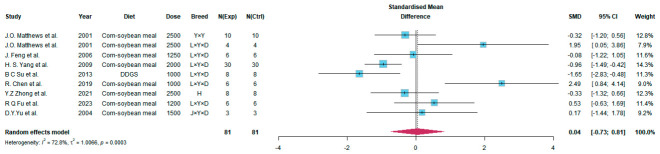
Forest plot of the overall effect of betaine supplementation on pork pH. The forest plots illustrate the subgroup analyses of betaine supplementation effects. The effect sizes are expressed as standardized mean differences (SMDs) with corresponding 95% confidence intervals (CIs). The size of each square is proportional to the study’s statistical weight. The solid vertical black line represents an SMD of zero (no effect). Points to the left of the line represent a reduction in the outcome metric, while points to the right indicate an increase. The overall pooled effect size for each subgroup and its 95% CI are indicated by the diamonds at the bottom. The blue squares represent the effect sizes of individual studies, and the red diamonds represent the pooled effect sizes [15,16,21,24,27,29,38,39,40].

**Figure A14:**
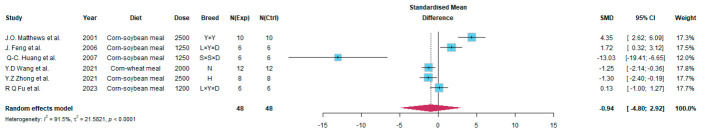
Forest plot of the overall effect of betaine supplementation on pig backfat thickness. The forest plots illustrate the subgroup analyses of betaine supplementation effects. The effect sizes are expressed as standardized mean differences (SMDs) with corresponding 95% confidence intervals (CIs). The size of each square is proportional to the study’s statistical weight. The solid vertical black line represents an SMD of zero (no effect). Points to the left of the line represent a reduction in the outcome metric, while points to the right indicate an increase. The overall pooled effect size for each subgroup and its 95% CI are indicated by the diamonds at the bottom. The blue squares represent the effect sizes of individual studies, and the red diamonds represent the pooled effect sizes [15,21,22,29,34,39].

Furthermore, the muscle shear force was significantly decreased [SMD = −2.63; 95% CI: (−3.51, −1.76); *p* < 0.05]. The accompanying heterogeneity was negligible (I^2^ = 0%), confirming the consistent positive impact of betaine on meat tenderness across the included studies. Conversely, the meta-analysis revealed that betaine treatment did not significantly affect the backfat thickness, meat color parameters (L*, a*, b*), or pH values (*p* > 0.05) (Figure A12, Figure A13 and Figure A14). These parameters were characterized by substantial inter-study heterogeneity (I^2^ > 75%), suggesting that the effects of betaine on these traits may be inconsistent or significantly confounded by variables such as breed and slaughter weight.

### 3.7. Analysis of Publication Bias

**Figure A15:**
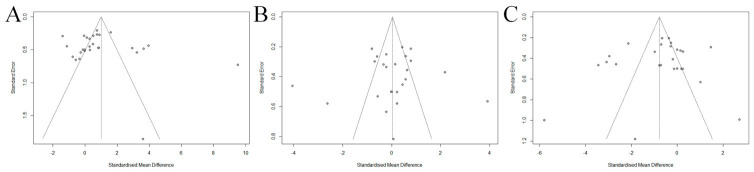
Funnel plots assessing publication bias regarding pig growth performance. (**A**) Average daily gain (ADG); (**B**) average daily feed intake (ADFI); (**C**) feed conversion ratio (FCR). The x-axis displays the effect size, expressed as the standardized mean difference (SMD), and the y-axis represents the standard error. Each dot represents an individual study included in the analysis. The symmetrical distribution of the studies around the vertical dotted line indicates the absence of significant publication bias.

**Figure A16:**
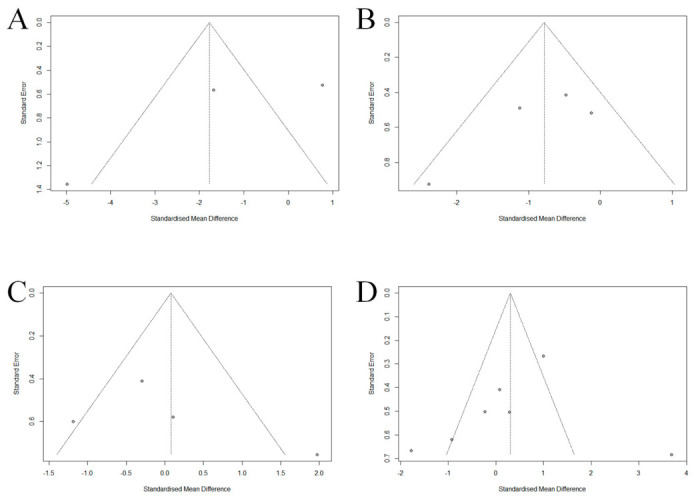
Funnel plots assessing publication bias regarding pig serum protein profiles. (**A**) Serum urea nitrogen (SUN); (**B**) urea; (**C**) albumin; (**D**) total protein. The x-axis displays the effect size, expressed as the standardized mean difference (SMD), and the y-axis represents the standard error. Each dot represents an individual study included in the analysis. The symmetrical distribution of the studies around the vertical dotted line indicates the absence of significant publication bias.

**Figure A17:**
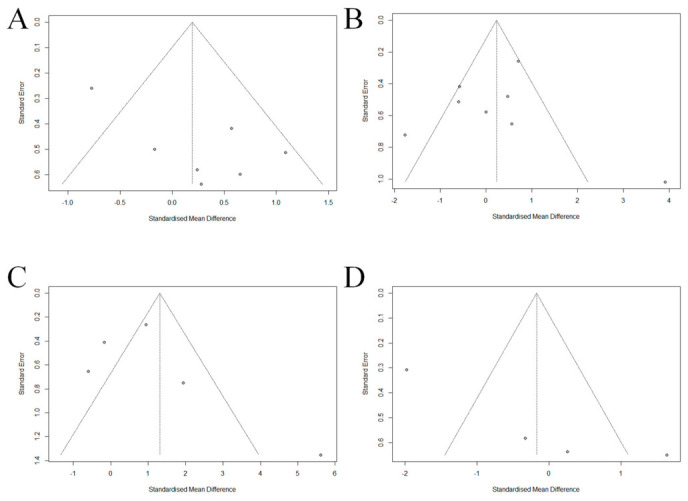
Funnel plots assessing publication bias regarding pig serum lipid profiles. (**A**) Total cholesterol (TC); (**B**) triglycerides (TGs); (**C**) high-density lipoprotein (HDL); (**D**) low-density lipoprotein (LDL). The x-axis displays the effect size, expressed as the standardized mean difference (SMD), and the y-axis represents the standard error. Each dot represents an individual study included in the analysis. The symmetrical distribution of the studies around the vertical dotted line indicates the absence of significant publication bias.

**Figure A18:**
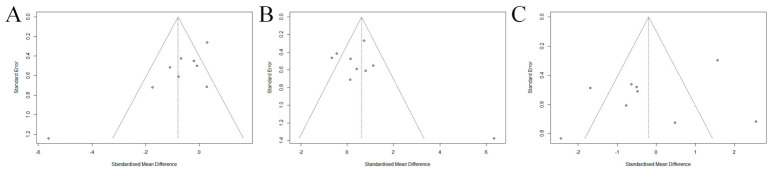
Funnel plots assessing publication bias regarding meat color parameters. (**A**) L*; (**B**) a*; (**C**) b*. The x-axis displays the effect size, expressed as the standardized mean difference (SMD), and the y-axis represents the standard error. Each dot represents an individual study included in the analysis. The symmetrical distribution of the studies around the vertical dotted line indicates the absence of significant publication bias.

**Figure A19:**
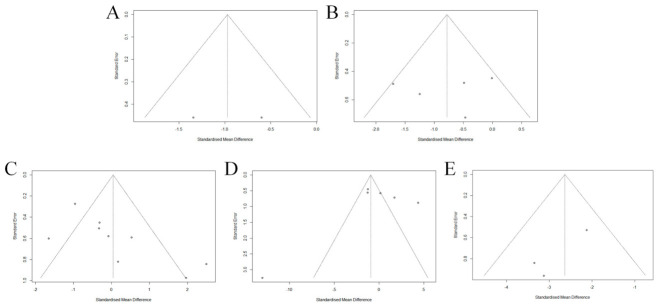
Funnel plots assessing publication bias for pork carcass traits. (**A**) Drip loss; (**B**) cooking loss; (**C**) pH; (**D**) backfat thickness; (**E**) shear force. The x-axis displays the effect size, expressed as the standardized mean difference (SMD), and the y-axis represents the standard error. Each dot represents an individual study included in the analysis. The symmetrical distribution of the studies around the vertical dotted line indicates the absence of significant publication bias.

Visual inspection of the funnel plots for growth performance (ADG, ADFI, and FCR) revealed a roughly symmetrical distribution of data points around the pooled effect sizes (Figure A15, Figure A16, Figure A17, Figure A18 and Figure A19). This observation was statistically confirmed by Egger’s regression test and Begg’s rank correlation test (*p* > 0.05), indicating no significant publication bias within the growth performance dataset (Table 8).

Regarding the serum metabolites and carcass traits, a quantitative bias assessment was limited by the limited number of included studies (predominantly n < 10). Nevertheless, visual inspection of the funnel plots showed no evidence of significant asymmetry.

## 4. Discussion

Betaine, acting as a pivotal methyl donor, plays a critical role in animal production by facilitating the biosynthesis of carnitine, creatine, and methylated amino acids [6]. Its efficacy in the practical rearing of ruminants and poultry has been well substantiated by prior investigations [41,42]. In the present study, a quantitative meta-analysis demonstrated that dietary betaine supplementation significantly improved growth performance, specific serum metabolites, and carcass traits of swine, although substantial heterogeneity was observed across certain parameters. Consequently, further investigation via a meta-regression elucidated that these results were subject to complex modulation by variables such as breed and dose. These findings lay a robust theoretical foundation for the application of betaine to optimize feed efficiency in swine husbandry.

Dietary betaine supplementation significantly enhanced the ADG and FCR of swine, while the ADFI remained unaltered. This observation suggests that the growth-promoting efficacy of betaine is not driven by the stimulation of appetite, but rather stems from the optimization of nutrient digestion, absorption, and metabolic utilization. Acting as a potent organic osmolyte, betaine serves to maintain the integrity of the intestinal epithelium, thereby safeguarding transmembrane nutrient transport. Furthermore, it has been documented to improve intestinal morphology and increase digestive enzyme activity [36,43,44]. Given that the gut constitutes the primary site for nutrient assimilation, betaine supplementation facilitates nutrient enzymatic digestion in the small intestine and enhances beneficial bacterial fermentation of dietary fiber in the large intestine [45]. These mechanisms collectively underpin the improvement in feed conversion efficiency without the prerequisite of increased feed intake.

Both the meta-regression and subgroup analyses indicated that the substantial heterogeneity observed in growth performance was predominantly attributed to variances in the animal breed and growth stage. Regarding genetic background, the regression analysis confirmed that breed constitutes the primary source of variance in ADG. Swine with divergent genetic backgrounds exhibited significant variability in betaine utilization efficiency, likely stemming from inherent differences in methyl metabolic capacity and protein accretion rates. Specifically, fast-growing commercial breeds (e.g., Duroc, Yorkshire) may possess elevated requirements for methyl donors to support their rapid growth rates. Conversely, Chinese indigenous breeds (e.g., Huanjiangxiang pigs) may exhibit heightened sensitivity to betaine, potentially attributable to specific gene polymorphisms or differential metabolic enzyme activities [46,47]. Regarding growth stage, although our statistical outcomes did not support a significant increase in feed intake during the piglet phase (*p* > 0.05), the developmental stage nonetheless accounted for a portion of the observed heterogeneity. This phenomenon is likely associated with the immature intestinal development and weaning stress characteristic of piglets. Betaine, a derivative of glycine, possesses specific palatability characteristics [44]. When combined with a protective role in maintaining intestinal barrier function under weaning stress [36,48], betaine may function to sustain—rather than significantly increase—feed intake under specific stress conditions. In contrast, during the growing–finishing phase, feed intake was predominantly regulated by energy homeostasis, which explains why the ADFI generally remained stable during this period.

Contrary to the conventional assumption of a simple “linear relationship” between betaine dose and growth performance, our subgroup and regression analyses delineated a distinct “threshold effect” with regard to the FCR. While the meta-regression failed to establish a linear correlation between the dose and FCR, the subgroup analysis identified 1250 mg/kg as the optimal inflection point for efficacy. Notably, the beneficial effects were attenuated at elevated doses (>2000 mg/kg).

Mechanistically, as a methyl donor, betaine metabolism is predominantly governed by the enzyme betaine-homocysteine methyltransferase (BHMT) [49]. Feng et al. [21] observed that moderate betaine supplementation significantly upregulated BHMT activity. We hypothesize that this phenomenon adheres to the principles of enzyme saturation kinetics. Once the exogenous supply of betaine satisfies the systemic demand for methyl groups, BHMT activity likely reaches a saturation point. Consequently, surplus betaine ceases to be utilized effectively and may conversely impose a metabolic burden, necessitating additional energy expenditure for its metabolic clearance and excretion [50].

The meta-analysis of serum biochemistry revealed that dietary betaine supplementation significantly decreased serum urea levels. Serum urea nitrogen (SUN) serves as a pivotal biomarker reflecting the rate of whole-body protein catabolism and amino acid oxidation. Although the reduction in SUN did not reach statistical significance, the direction of the effect size was consistent with that of urea (SMD < 0). Given the biochemical linearity between urea and SUN, the lack of statistical significance for SUN is likely attributable to the limited number of included studies reporting this specific parameter, which reduced the statistical power. Collectively, the results substantiate the hypothesis that betaine improves nitrogen retention. The observed reduction in serum urea suggests that betaine, by functioning as a methyl donor, probably enhances the efficiency of amino acid conversion and exerts a methionine-sparing effect, thereby optimizing overall protein utilization [44]. This increase in nitrogen deposition is likely intrinsically linked to the significant improvements observed in the ADG and FCR.

Regarding carcass traits, the meta-analysis demonstrated a significant reduction in muscle shear force, along with decreases in drip loss and cooking loss. We postulate that these improvements in tenderness and water-holding capacity (WHC) are attributed to the role of betaine as an organic osmolyte. By maintaining cellular homeostasis under osmotic stress, betaine preserves the structural integrity of the cell membrane [43]. The retention of intramuscular water not only contributes to enhanced juiciness but also substantially augments meat tenderness by preserving the structural architecture of myofibers [51].

In contrast, no statistically significant alterations were observed regarding serum lipids (TG, HDL, LDL) or carcass adipose traits (backfat thickness). Notably, these parameters were characterized by pronounced heterogeneity, diverging from a subset of the literature reporting that betaine significantly reduced backfat thickness. Taking these findings together with our meta-regression results, we postulate that the modulatory impact of betaine on lipid metabolism is highly dependent on the genetic background of the swine. Specifically, distinct mechanistic disparities in the utilization of methyl donors for lipid metabolism likely exist between lean-type and fatty-type genotypes. A sensitivity analysis revealed that the observed inter-study heterogeneity was predominantly driven by five specific outliers, attributable to variations in the experimental models and husbandry backgrounds. On one hand, specific genetic backgrounds or dietary conditions amplified the efficacy of betaine. Wang et al. [34] highlighted the heightened sensitivity of transcriptional regulation regarding lipid metabolism in Chinese indigenous fatty breeds (e.g., Ningxiang pigs). Huang et al. [22] demonstrated that moderate betaine supplementation could elicit significant growth-promoting effects via the activation of the growth hormone axis. Su et al. [38] proposed that within DDGS-based diets, betaine significantly improved meat quality through antioxidant mechanisms. On the other hand, specific feeding regimes or nutrient interactions obscured the independent effects of betaine. Urbańczyk et al. [17] failed to observe a singular therapeutic effect on blood lipids. This was likely due to complex interactions between the dietary energy concentration and chromium, which confounded the lipid-lowering function of betaine. Similarly, Fernández-Fígares et al. [37] indicated that a restricted feeding model might suppress the anticipated benefits of betaine, thereby contributing to the inconsistency of results across studies.

It is imperative to acknowledge certain limitations inherent in the present study. The substantial heterogeneity observed in results for growth performance, serum biochemical indices, and carcass traits likely stemmed from a multifactorial interplay of confounding variables, categorized as follows: (1) Biological Variability: Discrepancies in sex and baseline health status existed among the subject populations. Furthermore, due to the paucity of literature regarding specific breeds, certain genotypes were aggregated into the broader category of “indigenous breeds” during the subgroup analysis, potentially obscuring breed-specific nuances. (2) Intervention Heterogeneity: Variations in the purity, source, and supplementation duration of betaine were evident across studies. Notably, a subset of the included literature failed to explicitly report the purity specifications of the betaine utilized. (3) Environmental and Management Divergence: Global variations in husbandry practices—encompassing dietary formulations, stocking densities, and seasonal environmental conditions—introduced inevitable variability. Specifically, differences in the basal dietary methyl donor content, energy level, and fiber composition may have interacted with betaine, thereby modulating its overall efficacy. (4) Statistical Estimation: In instances where the data were reported exclusively as standard error of the mean (SEM), the algorithmic conversion to standard deviation (SD) may have introduced minor estimation errors. Extracting a single representative dose avoided control group statistical inflation, but inherently limited our evaluation of intra-study dose–response gradients. While our subgroup analysis successfully identified the breed and growth stage as pivotal determinants contributing to the heterogeneity in growth performance, the scarcity of reported data regarding specific serum metabolites and carcass traits constrained our capacity to conduct comprehensive subgroup or meta-regression analyses of these parameters. This data deficiency constitutes a primary limitation of the current work and highlights the need for more standardized experimental designs and rigorous data reporting in future investigations. Additionally, the scope of this study was restricted to evaluating the impact of betaine supplementation and dose specifically on growth performance across different breeds and stages; the dose–response relationship regarding serum biochemistry and carcass traits across varying physiological stages was not systematically explored. Future research should integrate dose thresholds with a cost–benefit analysis to evaluate the net economic returns of betaine supplementation in practical production, thereby guiding producers toward maximizing both biological and financial efficiency. Notwithstanding these limitations, this study provides substantial empirical evidence elucidating the precise effects of betaine on swine production.

## 5. Conclusions

In summary, this meta-analysis confirms that dietary betaine supplementation significantly improves swine growth performance. This improvement is mainly driven by enhanced feed conversion efficiency and protein accretion, alongside increased meat tenderness and water-holding capacity. Furthermore, the dose–response analysis reveals a distinct threshold effect, identifying 1250 mg/kg as the optimal supplementation dose for improving the feed conversion ratio (FCR), whereas this efficacy is attenuated at higher inclusion levels.

## Figures and Tables

**Table 1:** Search strategy.

Search	Query	Items Found
PubMed		
#1	(pig [all fields]) OR (swine [all fields]) OR (boar [all fields]) OR (piglet [all fields]) OR (sow [all fields]) OR (gilt [all fields]) OR (barrow [all fields])	382,944
#2	(betaine [all fields])	10,949
#1 AND #2		225
ScienceDirect		
#1	Title, abstract, keywords: (pig) OR (swine) OR (boar) OR (piglet) OR (sow) OR (gilt) OR (barrow);	9,333,604
#2	title, abstract, keyword: betaine	43,628
#1 AND #2		369
Web of Science		
#1	(pig [all fields]) OR (swine [all fields]) OR (boar [all fields]) OR (piglet [all fields]) OR (sow [all fields]) OR (gilt [all fields]) OR (barrow [all fields])	554,443
#2	(betaine [all fields])	20,252
#1 AND #2		259

Detailed search syntax and the corresponding number of records retrieved from PubMed, ScienceDirect, and Web of Science. The literature search encompassed publications between January 2001 and December 2025.

**Table 2:** Characteristics of the included studies.

No.	First Author	Year	Diet Type	Dose/(mg/kg)	Breed ^(1)^	Growth Stage	Initial BW or Days	Final BW or Duration	Result Indicators ^(2)^
1	J.O. Matthews et al. [14]	2001	Corn–soybean meal	500	Y × Y	Weaned piglets	5.5 kg	14.1 kg	①②③
2	J.O. Matthews et al. [15].	2001	Corn–soybean meal	2500	Y × Y	Growth and fattening period	51 kg	109 kg	①②③⑩⑫⑬⑭⑮⑯⑱⑲
3	J.O. Matthews et al. [16]	2001	Corn–soybean meal	2500	L × Y × D	Growth and fattening period	69 kg	115 kg	①②③④⑥⑦⑧⑨⑩⑫⑬⑭⑮⑲
4	J. Urbanczyk et al. [17]	2001	Corn–soybean meal	2000	L × Y	Growth and fattening period	30 kg	113 kg	①②③④⑤⑥⑦⑧
5	I. Fernandez-Figares et al. [18].	2002	Corn–soybean meal	1250	L × Y	Growth and fattening period	36 kg	64 kg	①②③⑩
6	B.V. Lawrence et al. [19]	2002	Corn–soybean meal	1250	Y × Y	Growth and fattening period	82.5 kg	116 kg	①②③
7	H. Siljander-Rasi et al. [10]	2003	Corn–soybean meal	500	L × Y	Growth and fattening period	29.6 kg	100 kg	①②③
8	D. Wray-Cahen et al. [20]	2004	Corn–soybean meal	1250	L × Y	Growth and fattening period	40 kg	11.6 d	①②③
9	J. Feng et al. [21]	2006	Corn–soybean meal	1250	L × Y × D	Growth and fattening period	62.5 kg	92.5 kg	①②③⑮⑯
10	Q.C. Huang et al. [22]	2007	Corn–soybean meal	1250	S × S × D	Growth and fattening period	55.7 kg	90 kg	①②③⑥⑨⑪⑯
11	F.R. Dunshea et al. [23]	2009	Wheat–canola meal	1500	L × Y	Growth and fattening period	59.5 kg	35 d	①③
12	H.S. Yang et al. [24]	2009	Corn–soybean meal	2000	L × Y × D	Growth and fattening period	65 kg	100 kg	①②③⑫⑬⑭⑮
13	J. Nakev et al. [12]	2009	Corn–soybean meal	1000	DW × L	Growth and fattening period	77.8 kg	81.8 d	①
14	I. Fernandez-Figares et al. [25]	2008	Barley–soybean meal	5000	AL	Growth and fattening period	20 kg	51.1 kg	①②③
15	L.S. Wang et al. [26]	2015	DDGS	1000	D	Growth and fattening period	60 kg	42 d	①②③
16	R. Chen et al. [27]	2019	Corn–soybean meal	1000	L × Y × D	Growth and fattening period	69 kg	120 kg	①②③⑫⑬⑭⑮
17	Y.T. Cheng et al. [28]	2021	Corn–soybean meal	2500	BM	Weaned piglets	35 d	125 d	①②③
18	Y.Z. Zhong et al. [29]	2021	Corn–soybean meal	2500	H	Growth and fattening period	10.55 kg	70 kg	①②③⑥⑨⑪⑫⑬⑭⑮⑯⑰⑲
19	M.H. Soares et al. [30]	2022	Corn–soybean meal	2500	L × Y × D	Growth and fattening period	89 kg	45 d	①②③⑦⑧⑪⑫⑬⑭⑲
20	F. Liu et al. [31]	2023	Corn–soybean meal	1000	L × Y × D	Growth and fattening period	26.6 kg	60.8 kg	①②③
21	D. Suste et al. [32]	2004	Wheat–pea meal	1500	L × Y	Growth and fattening period	60 kg	35 d	①②③
22	M. Lothong et al. [33]	2016	Corn–soybean meal	1250	L × Y × D	Growth and fattening period	74 kg	110 kg	①②③
23	Lima et al. [11]	2025	Corn–soybean meal	1000	L × Y × D	Growth and fattening period	29.51 kg	28 d	①②③④⑤⑥⑦⑧⑨
24	Y.D. Wang et al. [34]	2021	Corn–soybean meal	2000	N	Growth and fattening period	43.6 kg	81 d	①②③⑦⑧⑫⑬⑭⑯⑰⑱⑲
25	S.M. Mendoza et al. [13]	2017	DDGS	1500	L × Y × D	Growth and fattening period	39 kg	28 d	①②③⑥⑦⑧
26	J.M. Martins et al. [35]	2010	——	1000	AL	Growth and fattening period	36.7 kg	100 kg	①④⑤⑥
27	H.C. Wang et al. [36]	2020	Corn–soybean meal	1250	L × Y × D	Weaned piglets	8.52 kg	30 d	①②③
28	I. Fernandez-Figares et al. [37]	2012	Barley–soybean meal	5000	I	Growth and fattening period	69 kg	115 kg	④⑤⑦⑧⑩
29	B.C. Su et al. [38]	2013	DDGS	1000	L × Y × D	Growth and fattening period	60 kg	42 d	⑫⑬⑭⑮
30	R.Q. Fu et al. [39]	2023	Corn–soybean meal	1200	L × Y × D	Growth and fattening period	24.68 kg	119 d	⑫⑬⑭⑮⑯⑰
31	D.Y. Yu et al. [40]	2004	Corn–soybean meal	1500	J × Y × D	Growth and fattening period	20 kg	64 kg	①②③⑮

^(1)^ D: Duroc pig; L: Landrace Pig; Y: Yorkshire pig; BM: Bama miniature pig; I: Iberico pig; DW: Danube white pig; S: Segher pig; H: Huanjiangxiang pig; N: Ningxiang pig; AL: Alentejano pig; J: Jiaxing black pig; ——: not reported. ^(2)^ ① ADG: average daily gain; ② ADFI: average daily feed intake; ③ FCR: feed conversion ratio; ④ HDL: high-density lipoprotein; ⑤ LDL: low-density lipoprotein; ⑥ TP: total protein; ⑦ TC: total cholesterol; ⑧ TG: triglyceride; ⑨ ALB: albumin; ⑩ urea; ⑪ urea nitrogen; ⑫ meat color L*; ⑬ meat color a*; ⑭ meat color b*; ⑮ pH; ⑯ backfat thickness; ⑰ shear force; ⑱ drip loss; ⑲ cooking loss.

**Table 3:** Subgroup analysis of the effects of dietary betaine supplementation on average daily gain (ADG) in swine.

Item	*n*	SMD [95% CI]	*p*-Value	I^2^ (%)	*P*_Heterogeneity_	*P*_interaction_
ADG (Overall)		1.01 [0.23, 1.79]	0.011	93.3%	<0.001	
Phase						0.6582
Weaned piglets	3	1.35 [−0.12, 2.82]	0.071	90.4	<0.001	
Growth and fattening period	25	0.97 [0.10, 1.83]	0.029	93.7	<0.001	
Dose (mg/kg)						0.2984
500	2	0.59 [0.11, 1.08]	0.015	0	0.5762	
1000	6	0.21 [−0.52, 0.95]	0.570	83.7	<0.001	
1250	7	0.98 [−0.35, 2.31]	0.150	94.9	<0.001	
1500	4	0.15 [−0.39, 0.69]	0.586	24.1	0.2664	
2000	3	1.71 [−0.42, 3.88]	0.121	95.5	<0.001	
2500	5	2.46 [−1.17, 6.09]	0.183	97.8	<0.001	
Breed						0.1873
Y × Y	3	0.36 [−0.13, 0.85]	0.145	39.9	0.1892	
L × Y × D	10	0.16 [−0.45, 0.76]	0.607	89.9	<0.001	
L × Y	6	1.36 [−0.06, 2.78]	0.061	92.8	<0.001	
I B	9	2.09 [−0.04, 4.23]	0.056	95.8	<0.001	

ADG, average daily gain; n, number of included studies; SMD, standardized mean difference; CI, confidence interval; Y × Y, Yorkshire × Yorkshire; L × Y × D, Landrace × Yorkshire × Duroc; L × Y, Landrace × Yorkshire; I B, indigenous breeds. *p-*_value_ indicates the statistical significance of the pooled effect size; *P*_Heterogeneity_ denotes the significance of Cochran’s Q test for inter-study heterogeneity; *P*_interaction_ represents the statistical significance of the moderating effect across subgroups. I^2^ indicates the proportion of total variation across studies attributable to heterogeneity rather than chance.

**Table 4:** Subgroup analysis of the effects of dietary betaine supplementation on average daily feed intake (ADFI) in swine.

Item	*n*	SMD [95% CI]	*p*-Value	I^2^ (%)	*P*_Heterogeneity_	*P*_interaction_
ADFI (Overall)		0.03 [−0.51, 0.57]	0.9154	90.2%	<0.001	
Phase						0.0970
Weaned piglets	3	1.66 [−0.46, 3.78]	0.126	94.1	<0.001	
Growth and fattening period	22	−0.19 [−0.69, 0.31]	0.463	88.6	<0.001	
Dose (mg/kg)						0.2818
500	2	0.68 [0.20, 1.17]	0.006	0	0.5337	
1000	4	−0.16 [−0.76, 0.44]	0.606	68	0.0248	
1250	7	−0.20 [−1.61, 1.22]	0.785	95.4	<0.001	
1500	3	−0.01 [−0.63, 0.64]	0.993	0	0.9961	
2000	3	−0.15 [−0.75, 0.45]	0.624	64.2	0.0613	
2500	5	0.31 [−1.72, 2.33]	0.768	94.8	<0.001	
Breed						0.1004
Y × Y	3	−0.08 [−0.96, 0.80]	0.863	85.2	0.0012	
L × Y × D	10	−0.66 [−1.57, 0.24]	0.149	92.3	<0.001	
L × Y	5	0.07 [−0.36, 0.50]	0.741	33.5	0.1980	
I B	7	1.10 [0.04, 2.17]	0.043	86.9	<0.001	

ADFI, average daily feed intake; n, number of included studies; SMD, standardized mean difference; CI, confidence interval; Y × Y, Yorkshire × Yorkshire; L × Y × D, Landrace × Yorkshire × Duroc; L × Y, Landrace × Yorkshire; I B, indigenous breeds. *p*-value indicates the statistical significance of the pooled effect size; *P*_Heterogeneity_ denotes the significance of Cochran’s Q test for inter-study heterogeneity; *P*_interaction_ represents the statistical significance of the moderating effect across subgroups. I^2^ indicates the proportion of total variation across studies attributable to heterogeneity rather than chance.

**Table 5:** Subgroup analysis of the effects of dietary betaine supplementation on feed conversion ratio (FCR) in swine.

Item	*n*	SMD [95% CI]	*p*-Value	I^2^ (%)	*P*_Heterogeneity_	*P*_interaction_
FCR (Overall)		−0.78 [−1.38, −0.18]	0.0110	91.3%	<0.001	
Phase						0.2040
Weaned piglets	3	−0.32 [−0.78, 0.14]	0.168	55.9	0.1035	
Growth and fattening period	23	−0.86 [−1.54, −0.17]	0.015	92.1	<0.001	
Dose (mg/kg)						**0.0417**
500	2	−0.42 [−0.89, 0.06]	0.087	0%	0.3639	
1000	4	−0.34 [−1.66, 0.97]	0.608	93.1	<0.001	
1250	7	−1.30 [−2.63, 0.03]	0.056	95.7	<0.001	
1500	4	−0.24 [−0.88, 0.39]	0.448	32.9	0.2147	
2000	3	−1.14 [−2.91, 0.64]	0.209	94.8	<0.001	
2500	5	−1.22 [−3.23, 0.80]	0.237	89.7	<0.001	
Breed						0.8006
Y × Y	3	−1.54 [−3.40, 0.32]	0.104	94.1	<0.001	
L × Y × D	10	−0.85 [−2.08, 0.38]	0.175	94.7	<0.001	
L × Y	6	−0.73 [−1.53, 0.07]	0.073	80.4	<0.001	
I B	7	−0.42 [−1.65, 0.81]	0.507	88.6	<0.001	

FCR, feed conversion ratio; n, number of included studies; SMD, standardized mean difference; CI, confidence interval; Y × Y, Yorkshire × Yorkshire; L × Y × D, Landrace × Yorkshire × Duroc; L × Y, Landrace × Yorkshire; I B, indigenous breeds. *p*-value indicates the statistical significance of the pooled effect size; *P*_Heterogeneity_ denotes the significance of Cochran’s Q test for inter-study heterogeneity; *P*_interaction_ represents the statistical significance of the moderating effect across subgroups. I^2^ indicates the proportion of total variation across studies attributable to heterogeneity rather than chance. Bold values indicate statistical significance.

**Table 6:** Meta-regression analysis of the effects of betaine dose, breed, and growth phase on growth performance.

Outcome	Subgroup	Coefficient	95%CI	*P_regression_*
ADG, g/d	Phase	1.2886	[−2.1101, 2.9411]	0.7471
	Dose	0.0005	[−0.0006, 0.0012]	0.5357
	Breed	ST1	ST1	**0.0001**
ADFI, g/d	Phase	0.7748	[0.2675, 3.3046]	**0.0212**
	Dose	0.0003	[−0.0006, 0.0006]	0.9726
	Breed	ST1	ST1	**0.0457**
FCR, g/g	Phase	0.9510	[−1.2820, 2.4459]	0.5406
	Dose	0.0004	[−0.0004, 0.0011]	0.3171
	Breed	ST1	ST1	0.6295

ADG, average daily gain; ADFI, average daily feed intake; FCR, feed conversion ratio; CI, confidence interval. *P*_regression_ indicates the statistical significance of the covariate in the meta-regression model. For the multi-categorical variable “Breed”, the overall omnibus *p*-value is presented, and the symbol “ST1” indicates that the specific coefficients for each sub-breed are detailed in Table A1. Bold values indicate statistical significance.

**Table 7:** Effect of dietary betaine on serum metabolites, carcass traits and meat quality of swine.

	Item	*n*	SMD	95%CI	*p*-Value	I^2^/%	*p*-Value (Heterogeneity)
Serum Metabolites	HDL, mmol/L	5	1.31	[−0.57, 3.19]	0.172	84.2%	<0.001
	LDL, mmol/L	4	−0.17	[−1.68, 1.34]	0.824	90.5%	<0.001
	Albumin, g/dL	4	0.08	[−1.12, 1.27]	0.898	73.2%	0.010
	Triglycerides, mmol/L	7	−0.08	[−0.68, 0.52]	0.629	66.3%	0.007
	Total cholesterol, mmol/L	7	0.19	[−0.35, 0.74]	0.489	64.2%	0.010
	Total protein, g/L	7	0.30	[−0.92, 1.52]	0.627	86.8%	<0.001
	Urea N, mmol/L	3	−1.78	[−4.90, 1.35]	0.266	90.4%	<0.001
	Urea, mmol/L	4	−0.79	[−1.45, −0.12]	0.020	46.3%	0.133
Carcass Traits	pH	9	0.04	[−0.73, 0.81]	0.919	72.8%	<0.001
	Backfat thickness, mm	6	−0.94	[−4.80, 2.92]	0.633	91.5%	<0.001
	Drip loss, %	2	−0.97	[−1.70, −0.24]	0.009	23.7%	0.252
	Cooking loss, %	5	−0.78	[−1.43, −0.13]	0.018	48.5%	0.100
	a*	9	0.62	[−0.22, 1.45]	0.149	75.3%	<0.001
	b*	9	−0.20	[−1.17, 0.77]	0.692	88.0%	<0.001
	L*	9	−0.80	[−1.59, 0.00]	0.049	75%	<0.001
	Shear force, N	3	−2.63	[−3.51, −1.76]	0.000	0%	0.389

n, number of included studies; SMD, standardized mean difference; CI, confidence interval; HDL, high-density lipoprotein; LDL, low-density lipoprotein; Urea N, urea nitrogen; L*, lightness; a*, redness; b*, yellowness. The *p*-value indicates the statistical significance of the pooled effect size; the *p*-value (Heterogeneity) denotes the significance of Cochran’s Q test for inter-study heterogeneity. I^2^ indicates the proportion of total variation across studies attributable to heterogeneity rather than chance.

**Table 8:** Publication bias analysis of the ADG, ADFI and FCR studies.

Outcome	Egger	Begg
ADG	0.2952	0.8744
ADFI	0.8240	0.6404
FCR	0.4871	0.9122

ADG, average daily gain; ADFI, average daily feed intake; FCR, feed conversion ratio. The values in the “Egger” and “Begg” columns represent the *p*-values from Egger’s linear regression test and Begg’s rank correlation test, respectively. *p* > 0.05 indicates the absence of significant publication bias.

## Data Availability

No new data were created or analyzed in this study. Data sharing is not applicable to this article.

## Appendix A

### Appendix A.2

**Table A1:** Subgroup meta-regression analysis of swine breed effects on average daily gain (ADG), average daily feed intake (ADFI), and feed conversion ratio (FCR).

Outcome	Subgroup		Coefficient	95%CI	*P_regression_*
ADG, g/d	Breed	Overall			**0.0001**
		BM	1.2637	[0.3174, 5.2712]	**0.0270**
		D	1.2750	[−2.3376, 2.6604]	0.8993
		DW × L	1.3347	[−3.1064, 2.1255]	0.7133
		H	1.3791	[6.6457, 12.0519]	**0.0001**
		J × Y × D	2.1959	[−0.8468, 7.7611]	0.1154
		L × Y	0.6179	[0.0061, 2.4282]	**0.0489**
		AL	0.9659	[−2.4438, 1.3424]	0.6958
		N	1.2428	[−2.0884, 2.7832]	0.7798
		S × S × D	1.2671	[1.0209, 5.9880]	**0.0057**
		Y × Y	0.7684	[−1.2235, 1.7886]	0.3677
ADFI, g/d	Breed	Overall			**0.0457**
		BM	1.3000	[2.0192, 7.1150]	**0.0004**
		D	1.7660	[−3.0232, 3.8995]	0.4937
		H	1.2734	[−1.6242, 3.3675]	0.2272
		AL	1.3316	[−2.1764, 3.0435]	0.0807
		L × Y	0.6485	[−0.5583, 1.9836]	0.2718
		J × Y × D	1.4273	[−2.0828, 3.5121]	0.6166
		N	1.2427	[−1.2167, 3.6545]	0.3267
		S × S × D	1.2274	[0.4205, 5.2317]	**0.0213**
		Y × Y	0.7611	[−0.9215, 2.0620]	0.4537
FCR, g/g	Breed	Overall			0.6295
		BM	1.7081	[−2.3849, 4.3108]	0.5729
		D	1.7499	[−2.6052, 4.2542]	0.6375
		H	1.6895	[−2.8589, 3.7639]	0.7888
		J × Y × D	2.0497	[−5.0281, 3.0068]	0.6220
		L × Y	0.8547	[−1.5842, 1.7660]	0.9153
		AL	1.9483	[−0.2860, 7.3513]	0.1153
		N	1.7262	[−2.7558, 4.0107]	0.7162
		S × S × D	1.7329	[−5.6583, 1.1346]	0.1918
		Y × Y	1.0795	[−2.8302, 1.4015]	0.5082

ADG, average daily gain; ADFI, average daily feed intake; FCR, feed conversion ratio; CI, confidence interval. *P*_regression_ indicates the statistical significance of the covariate in the meta-regression model. D: Duroc pig; L: Landrace pig; Y: Yorkshire Pig; BM: Bama miniature pig; DW: Danube White pig; S: Segher Pig; H: Huanjiangxiang pig; N: Ningxiang pig; AL: Alentejano pig; J: Jiaxing black pig. Bold values indicate statistical significance.
